# Deep branch of the radial nerve: effect of pronation/supination on longitudinal nerve alignment

**DOI:** 10.1007/s00256-023-04332-5

**Published:** 2023-04-03

**Authors:** Aurea V.R. Mohana-Borges, Sergio A. L. Souza, Ronaldo Mohana-Borges, Sheronda Statum, Christine B. Chung

**Affiliations:** 1 Radiology, Federal University of Rio de Janeiro, Rio de Janeiro, Brazil; 2 Radiology, University of California, San Diego, USA; 3 Laboratory of Biotechnology and Structural Bioengineering, Biophysics Institute Carlos Chagas Filho, Rio de Janeiro Federal University, Rio de Janeiro, Brazil; 4 Radiology, Veterans Affairs Medical Center, San Diego, USA

**Keywords:** Ultrasound, High-resolution ultrasound, Nerve, Deep branch of the radial nerve, Posterior interosseous nerve, Supinator muscle, Arcade of Frohse

## Abstract

**Objective:**

To evaluate the effect of maximal pronation and supination of the forearm on the alignment and anatomic relationship of the deep branch of the radial nerve (DBRN) at the superior arcade of the supinator muscle (SASM) by using high-resolution ultrasound (HRUS).

**Materials and methods:**

In this cross-sectional study, HRUS in the long axis of the DBRN was performed in asymptomatic participants enrolled from March to August 2021. DBRN alignment was evaluated by measuring angles of the nerve in maximal pronation and maximal supination of the forearm independently by two musculoskeletal radiologists. Forearm range of motion and biometric measurements were recorded. Student *t*, Shapiro–Wilk, Pearson correlation, reliability analyses, and Kruskal–Wallis test were used.

**Results:**

The study population included 110 nerves from 55 asymptomatic participants (median age, 37.0 years; age range, 16–63 years; 29 [52.7%] women). There was a statistically significant difference between the DBRN angle in maximal supination and maximal pronation (Reader 1: 95% CI: 5.74, 8.21, *p* < 0.001, and Reader 2: 95% CI: 5.82, 8.37, *p* < 0.001). The mean difference between the angles in maximal supination and maximal pronation was approximately 7° for both readers. ICC was very good for intraobserver agreement (Reader1: *r* ≥ 0.92, *p* < 0.001; Reader 2: *r* ≥ 0.93, *p* < 0.001), as well as for interobserver agreement (phase 1: *r* ≥ 0.87, *p* < 0.001; phase 2: *r* ≥ 0.90, *p* < 0.001).

**Conclusion:**

The extremes of the rotational movement of the forearm affect the longitudinal morphology and anatomic relationships of the DBRN, primarily demonstrating the convergence of the nerve towards the SASM in maximal pronation and divergence in maximal supination.

## Introduction

The deep branch of the radial nerve (DBRN) is one of the major divisions of the radial nerve in the elbow region. DBRN entrapment occurs primarily at the level of the superior arcade of the supinator muscle (SASM), which is also known as the arcade of Frohse [1]. An association between DBRN entrapment, SASM, and repetitive rotational forearm movements has been previously proposed [1–4]. Paralytic and painful entrapment syndromes of the DBRN, i.e., posterior interosseous nerve (PIN) and radial tunnel syndromes, have been associated with sports activities and heavy manual work requiring forceful and repetitive forearm pronation and supination with the elbow extended [5]. PIN syndrome has been associated with sports, such as bodybuilding/weightlifting, frisbee, gymnastics, tennis/racquetball, and swimming [6], and has been reported in violinists, orchestral conductor, bartender, corsetiere, and dairy workers [7]. In addition, maximal pronation has been shown to increase pressure on the nerve beneath the SASM and the supinator in cadavers [5].

Several imaging methods can be applied to assess peripheral nerves. The role of ultrasonography has grown in the last decades with the advent of higher-frequency transducers and software improvements [8, 9]. High-resolution ultrasonography (HRUS) is now considered the first-line imaging modality for peripheral nerve evaluation because of, but not limited to, the high spatial resolution for visualization of tiny nerves, its cost, the ease of exam for the patient, and the capability of real-time dynamic images [10, 11]. However, a knowledge gap persists for several aspects of the neurodynamics of the DBRN by non-invasive imaging methods, including HRUS.

Our study aimed to evaluate how the extremes of rotational movement of the forearm affect the longitudinal alignment of the DBRN at the SASM by using HRUS. We investigated alterations in the long axis configuration of DBRN by measuring the angulation of the nerve. The hypothesis is that the DBRN has a more pronounced convergence towards the SASM when the forearm is in maximal pronation compared to maximal supination. We aim to understand the neurodynamics of the DBRN by imaging and to correlate the findings with the pathophysiology of the entrapment syndromes.

## Materials and methods

### Study design

Institutional Review Board approval was secured. Written informed consent was obtained from all participants. Participants were enrolled from March to August 2021, and 55 asymptomatic volunteers composed the final study population. The evaluation was performed bilaterally (55 subjects) in 110 limbs resulting in 110 nerves for the study.

### Participants

Inclusion criteria were asymptomatic volunteers more than 15 years of age, either gender. Exclusion criteria were as follows: (a) symptoms that compromise sustained forearm pronation and supination positions, (b) functional deficit in the forearm range of motion (ROM), (c) previous history of supinator tunnel surgery or DBRN decompression or hydrodissection, and (d) incidental masses compressing the nerve.

**Fig. 1:**
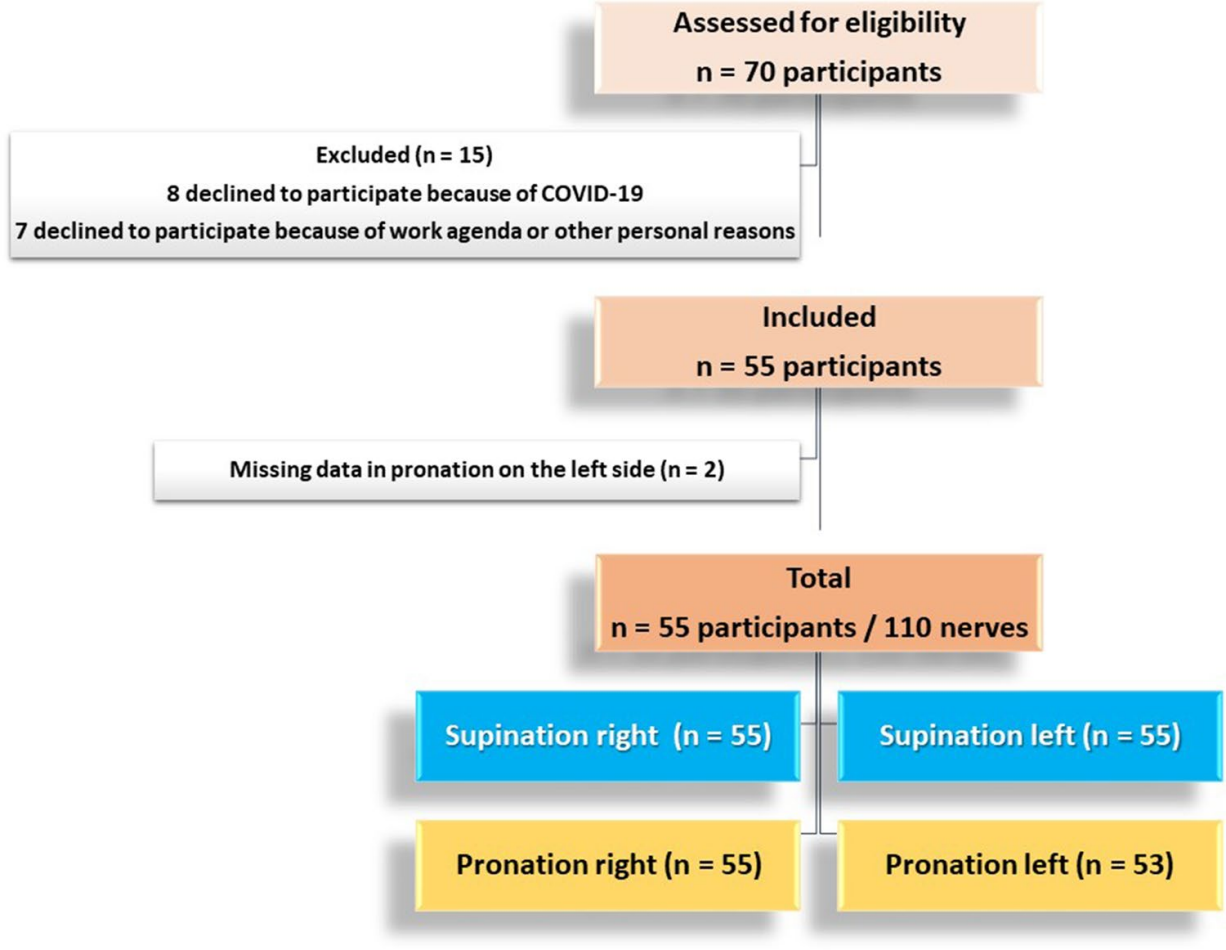
Flowchart of the study population

The forearm ROM was measured with an inclinometer (Sanny®) and considered normal if above 50° of pronation and 55° of supination [12]. A bioimpedance foot scale (Relaxmedic®) was used for measurements of weight, body mass index (BMI), percentage of muscle mass, and percentage of total body fat. BMI was divided into categories as follows: underweight ≤ 18.5, normal weight = 18.5–24.9, overweight = 25–29.9, obesity class I = 30–34.9, obesity class II = 35–39.9, and obesity class III ≥ 40. Hand dominance and height were recorded based on subjects’ reporting. The subjects were questioned about upper limb workouts, sports activities with the upper limb, hours of computer work, and forearm rotation during work. Figure 1 provides a flowchart of patient enrollment.

### HRUS technique

HRUS was performed with an 18-5 MHz linear transducer (Philips, Affiniti 50) by a single musculoskeletal radiologist (A.M.B) with more than 20 years of experience. During the examination, participants sat in a comfortable chair with their forearms extended and supported by a table which was adjusted to put the shoulder in the position of 90° of flexion. A straight splint surrounded by fixation straps was used on the participants’ hands and wrists for partial immobilization in the neutral position.

**Fig. 2:**
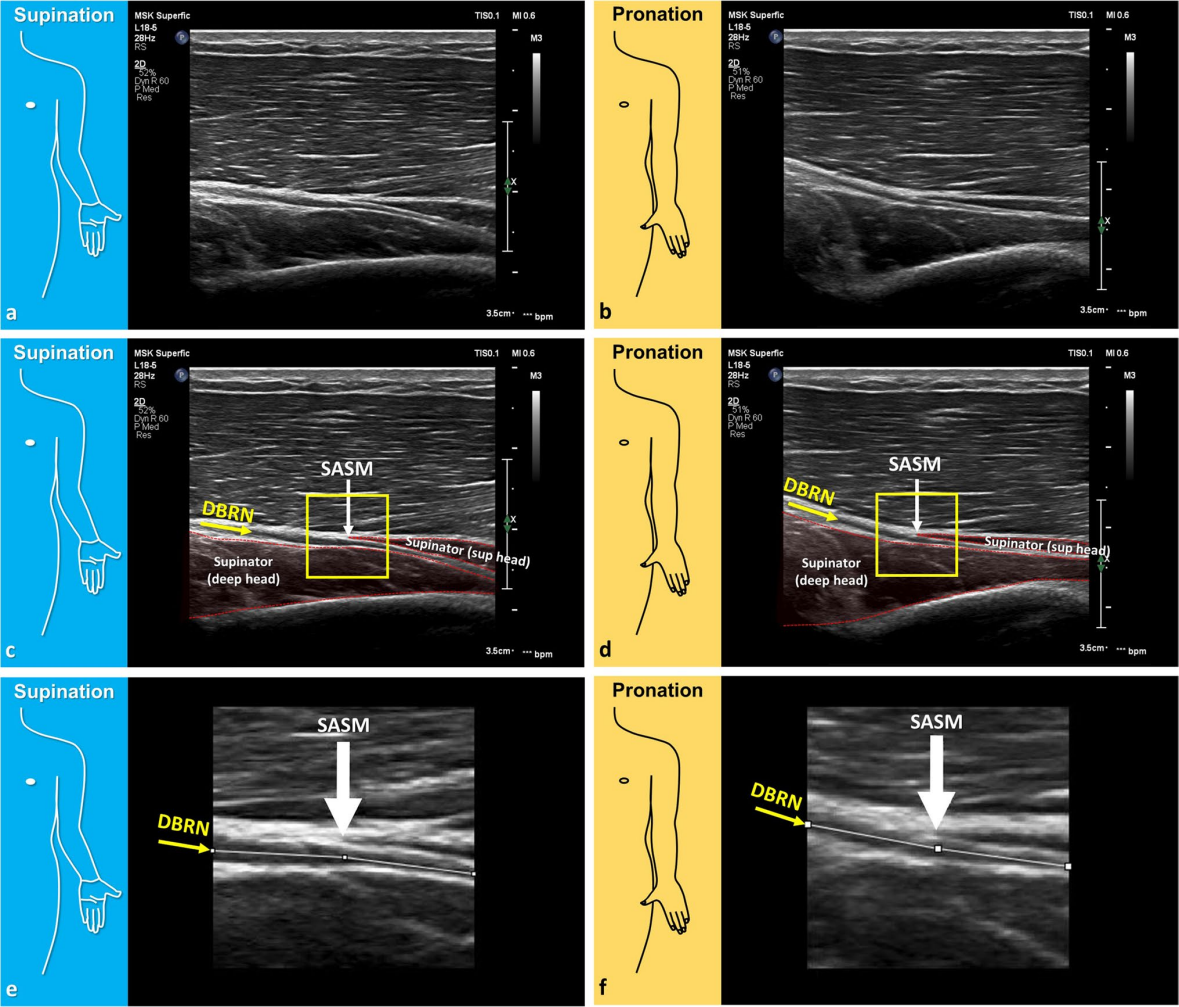
Reference image and ROI selection for measurement of the DBRN angle. HRUS in the long axis of the DBRN in a 53-year-old right-hand dominant male in forearm **(a, c, e)** supination and **(b, d, f)** pronation. **a, b** Reference image. **c, d** ROI selection. **e, f** Final ROI. The reference image was selected from a cine clip and considered the best for visualization of the longest segment of the nerve, above and below the superior arcade of the supinator muscle (SASM). Sup (deep head) = deep head of supinator muscle. Sup (sup head) = superficial head of supinator muscle

The radiologist selected the best image for DBRN angle measurements from a cine clip performed on the nerve’s long axis (Fig. 2). The reference image was the best for visualization of the longest segment of the nerve, proximal and distal to the SASM. Image selection and anonymization were performed offline. Regions of interest (ROIs) of 1 × 1 cm centered at the level of the SASM were prepared with the open-source Fiji ImageJ (64-bit version).

### Image analysis

Two musculoskeletal radiologists (A.M.B and C.B.C.) with more than 20 years of experience independently analyzed the reference images and measured the DBRN angles. The angle’s vertex was located at the center of the nerve at the SASM level, and the rays were traced as straight lines in the center of the long axis of the nerve.

A training session was performed on 30 images. Subsequently, angle measurements were obtained in two phases. In phase 1, measurements of the DBRN angle were acquired in four sets of images separated as supination right (SR), pronation right (PR), supination left (SL), and pronation left (PL). In phase 2, readers repeated the measurements in a single set of randomized images. Repeated measures were done after at least 15 days to mitigate recall bias. A researcher not involved in image analysis (RMB) was tasked to blind the sample set. Images were relabeled with random numbers generated in Excel with the RAND function followed by RANK. EQ to avoid repeating numbers.

### Angle interpretation

**Fig. 3:**
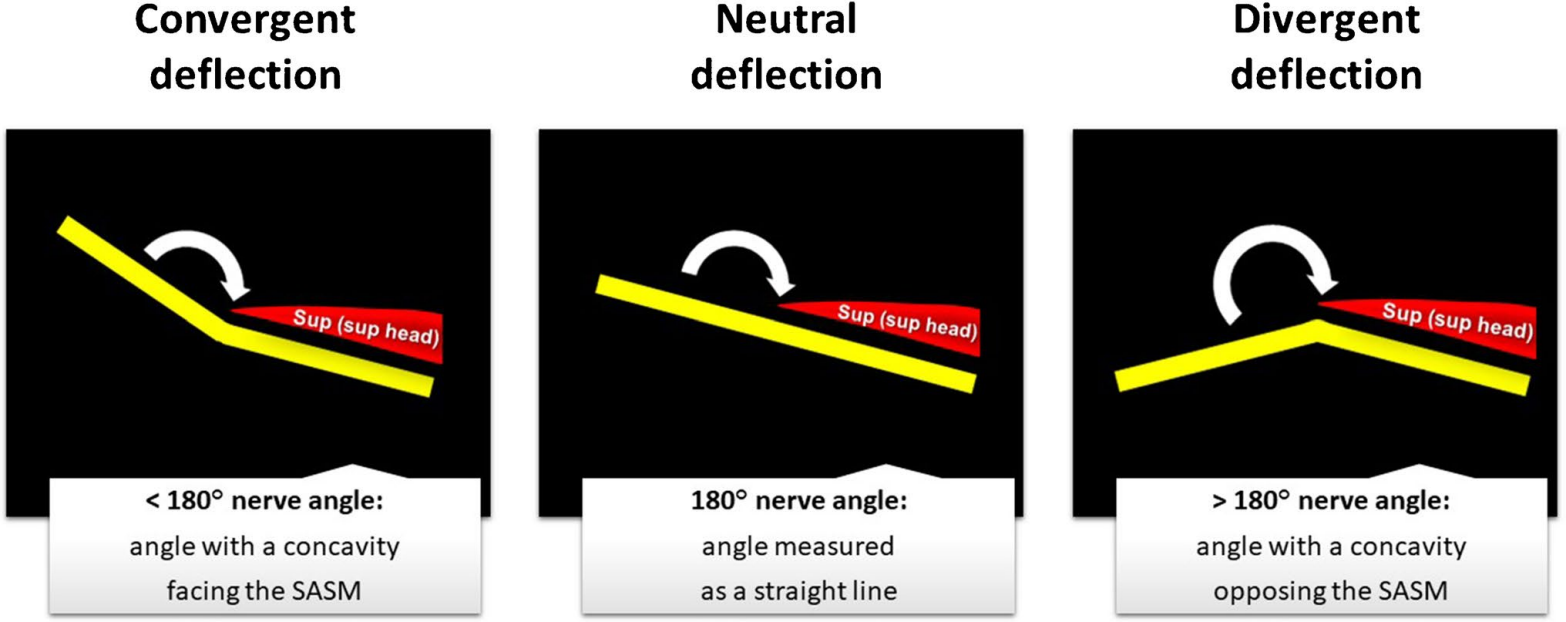
References for angle interpretation

References for angle interpretation were as follows (Fig. 3):

- 180° nerve angle (neutral deflection): angle measured as a straight line.
- < 180° nerve angle (convergent deflection): angle with a concavity facing the direction of the SASM.
- > 180° nerve angle (divergent deflection): angle with a concavity facing the opposite direction of the SASM.

### Statistical analysis

Statistical analysis and graphs were performed using the open-source Jamovi software (2.2.2 version) and Excel 365. The normality of the data was assessed by using the Shapiro–Wilk test. Parametric data were presented as the mean ± standard deviation, and non-parametric data as the median and interquartile range (IQR). Welch’s test was used to estimate the unequal variance of age and sex, the Bland–Altman plot to demonstrate agreement between readers, and Kruskal–Wallis test to correlate DBRN angles and upper limb activities. A paired *t*-test was used to compare the DBRN angles on different sides of the body and positions of the forearm. Two-tailed *P* < 0.05 was considered to indicate a statistically significant difference. The effect size (*d*) was classified as follows: very small = 0.01, small = 0.2, medium = 0.5, large = 0.8, very large = 1.2, and huge = 2. Pearson correlation and reliability analysis were used to determine inter and intraobserver agreements. Intraclass correlation coefficient (ICC) with a 95% confidence interval was classified as null (i.e., 0), slight (> 0 to ≤ 0.20), fair (> 0.21 to ≤ 0.40), moderate (> 0.41 to ≤ 0.60), good (> 0.61 to ≤ 0.80), and very good (> 0.81). ICC was based on a mean rating (*k* = 2), consistency agreement, and a 2-way fixed-effects model.

## Results

### Participant characteristics

Participant characteristics are listed in Table 1. The study population consisted of 110 nerves from 55 asymptomatic subjects (median age, 37.0 years; IQR, 23.5–51.0 years; age range, 16–63 years; 29 [52.7%] women). We found no significant difference in age between women (median 37 years, IQR, 24.0–50.0 years) and men (median 36.5 years, IQR, 22.8–52.8 years). Weight, height, BMI, percentage of muscle mass, percentage of total body fat, forearm length, and arm, elbow, and forearm circumferences had normal distribution. Limb and arm length and age had a distribution different from normality. One participant deferred having the weight measured. Among the other participants (*N* = 54), one (1.9%) was underweight, 19 (35.2%) were normal in weight, 16 (29.6%) were overweight, and 18 (33.3%) were obese. In the group with obesity (*N* = 17), the majority had obesity grade I (89.3%).

**Table 1:** Participant characteristics

Parameter	*N* (%)	Mean (±SD)	Median (IQR)	Shapiro–Wilk (*w* / *p*)
Age (y)	55 (100)	37.4 (±14.0)	37 (27.5)	0.925 / 0.002
Sex	55 (100)	…	…	…
Female	29 (52.7)	…	…	0.934 / 0.069
Male	26 (47.3)	…	…	0.911 / 0.027
Weight (kg)*	54 (98.2)	78.6 (±19.16)	…	0.959 / 0.064
Height (m)	55 (100)	1.7 (±0.08)	…	0.984 / 0.651
BMI (kg/m^2^)*	54 (98.2)	27.3 (±5.58)	…	0.967 / 0.147
Muscle mass**	53 (96.4)	35.2 (±5.07)	…	0.975 / 0.340
Total body fat**	53 (96.4)	30.3 (±9.18)	…	0.983 / 0.646
Hand dominance	55 (100)	…	…	…
Right	51 (92.7)	…	…	…
Left	3 (7.3)	…	…	…
Limb length (cm)	110 (100)	47.6 (±2.71)	47.5 (3.3)	0.974 / 0.032
Right	55 (50)	47.7 (±2.79)	…	0.972 / 0.233
Left	55 (50)	47.4 (±2.65)	…	0.972 / 0.223
Arm length (cm)	110 (100)	22.3 (±1.58)	22.3 (1.9)	0.973 / 0.023
Right	55 (50)	22.3 (±1.66)	…	0.979 / 0.453
Left	55 (50)	22.3 (±1.51)	22.5 (1.5)	0.954 / 0.036
Forearm length (cm)	110 (100)	25.2 (±1.87)	…	0.984 / 0.198
Right	55 (50)	25.4 (±1.89)	…	0.974 / 0.277
Left	55 (50)	25.1 (±1.85)	…	0.982 / 0.585
Arm circumference (cm)	110 (100)	32.0 (±4.73)	…	0.979 / 0.087
Right	55 (50)	31.8 (±4.81)	…	0.980 / 0.468
Left	55 (50)	32.1 (±4.70)	…	0.980 / 0.475
Elbow circumference (cm)	110 (100)	26.6 (±2.76)	…	0.982 / 0.141
Right	55 (50)	26.6 (±2.75)	…	0.983 / 0.644
Left	55 (50)	26.6 (±2.80)	…	0.979 / 0.428
Forearm circumference (cm)	110 (100)	26.4 (±3.00)	…	0.987 / 0.377
Right	55 (50)	26.6 (±3.00)	…	0.987 / 0.825
Left	55 (50)	26.2 (±3.01)	…	0.982 / 0.598
Supination right limb	54 (98.2)	84.6 (±8.48)	85.0 (9.7)	0.905 / < 0.001
Pronation right limb	54 (98.2)	82.3 (±7.88)	…	0.962 / 0.082
Supination left limb	54 (98.2)	84.4 (±10.55)	85.5 (10)	0.945 / 0.015
Pronation left limb	54 (98.2)	82.7 (±6.12)	…	0.977 / 0.387

Note. –. *One participant deferred weight data point acquisition and did not have the range of motion recorded. **In one additional participant, the scale calculated the subcutaneous fat but was unable to calculate muscle mass and total body fat

There were no statistically significant differences in the forearm total ROM comparing the right and left sides of the body, as well as comparing the positions of SR-SL, PR-PL, SR-PR, and SL-PL. The right hand was dominant in 51 of 55 participants (92.7%). Rotational movements at work were reported by 14 out of 55 participants (25.4%). Among them (*N* = 14), 7 (50%) were enrolled in laboratory activities, 5 (35.7%) in work without the use of machinery, and 2 (14.3%) in home activities. On average, participants had 4.7 h (0–13 h) of computer work. Upper limb sports activity (in the past or in the present) was reported by 34 out of 55 participants (61.8%). The sports were swimming (*N* = 24), volleyball (*N* = 8), basketball (*N* = 4), handball (*N* = 1), and martial arts (*N* = 13). Among these 34 participants, 13 (38.2%) were simultaneously engaged in more than one sport activity and/or martial arts.

One participant did not have the range of motion recorded but had a normal range of motion during physical and ultrasound examinations. In one additional participant, the scale calculated the subcutaneous fat but could not calculate the percentage of muscle mass and total body fat because of obesity class 3. Because of time constraints, two participants did not complete the evaluation in pronation on the left side. These missing data were statistically treated by excluding cases analysis by analysis.

### Analysis of the DBRN Angle

**Fig. 4:**
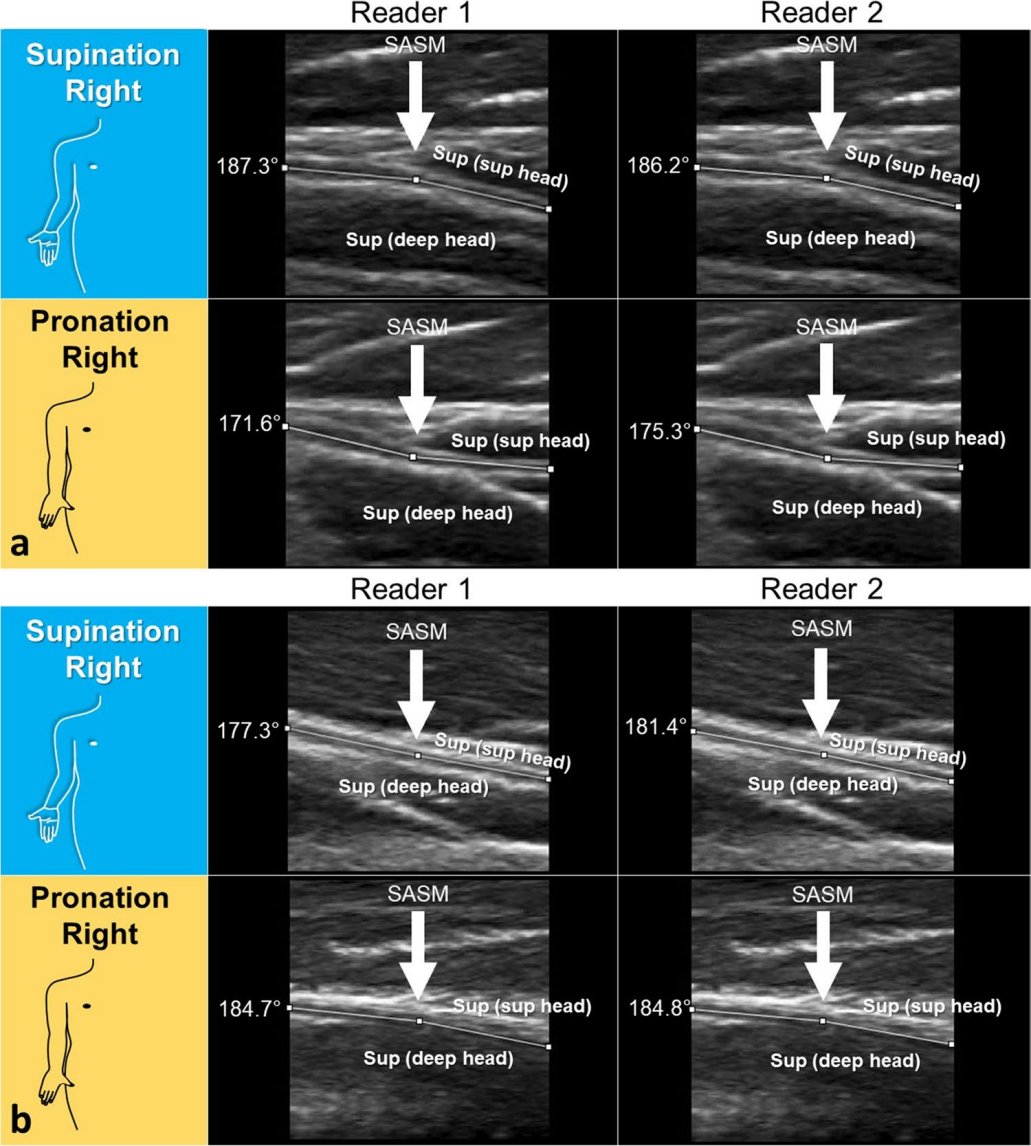
DBRN angle at the level of the superior arcade of the supinator muscle (SASM) with comparison between readers. **a** HRUS in the long axis of DBRN in a 38-year-old woman right-handed. Observe in supination that the angle is greater than 180° (arrows), corresponding to a divergent deflection. In pronation, the angle is less than 180°, corresponding to a convergent deflection. This case demonstrates the most common pattern of angulation of the nerve. Angle difference: 15.7 (reader 1), 10.9 (reader 2). **b** HRUS in the long axis of DBRN in a 22-year-old woman left-handed. This case demonstrates the less frequent pattern of nerve angulation in pronation with the divergence of the nerve from the SASM. Note that in supination, reader 1 measures a convergent angle and reader 2 to a divergent angle. Angle difference: −7.4 (reader 1), −3.4 (reader 2). Sup (sup head) = superficial head of the supinator muscle. Sup (deep head) = deep head of supinator muscle

**Fig. 5:**
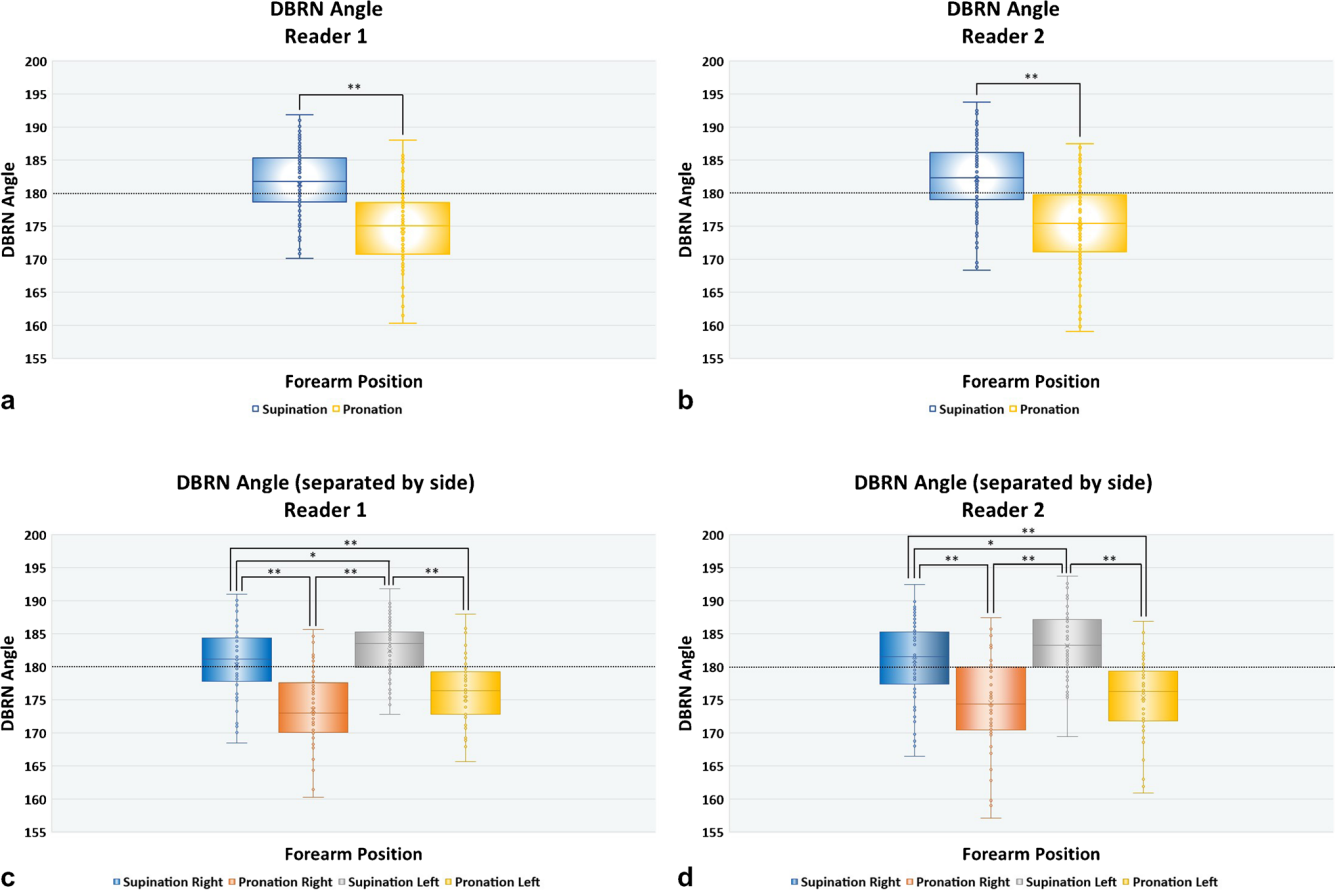
Box and whisker graph of DBRN angles at the level of the SASM according to **(**a, b) position of the forearm and **(**c, d) side and position of the forearm. Angles below 180° (dotted line) correspond to a convergent deflection relative to the SASM. Angles above 180° correspond to a divergent deflection relative to the SASM. Note higher angles in the supinated position and lower angles in the pronated position. The mean value for the pronated position is below 180° and for the supinated position above 180° for both readers. The line across the box denotes the median value, while the box boundaries represent the 25th and 75th percentiles. X corresponds to the mean value. Whiskers indicate the minimum and maximum values

There was a statistically significant difference in DBRN angle in maximal supination and maximal pronation of the forearm (Reader 1: 95% CI: 5.74, 8.21, *p* < 0.001, *d* = 1.08, and Reader 2: 95% CI: 5.82, 8.37, *p* < 0.001, *d* = 1.29) (Figs. 4 and 5). The mean difference between the angles was approximately 7.0° for both readers (Reader 1, mean: 6.98°, range ≥ - 14.2° ≤ + 29.6°, IQR: 3.5–10.8°; and Reader 2, mean: 7.09°, range ≥ - 12.2° ≤ + 35.3°, IQR: 2.9–11.1°). In addition, there was a statistically significant difference between the DBRN angles for the pairwise comparisons of SR-PR, SL-PL, SR-PL, and SL-PR (*p* < 0.001) for both readers (Tables 2 and 3). Comparison of SR-SL resulted in a statistically significant difference (Reader 1, *p* = 0.022; Reader 2, *p* = 0.020) but with a small negative effect size (Reader 1, *d* = −0.3, Reader 2, *d* = −0.3). Comparison of PR-PL resulted in no statistically significant difference for both readers (Reader 1, *p* = 0.058; Reader 2, *p* = 0.431).

**Table 2:** Comparisons of means of DBRN angle in different sides and positions of the forearm for Reader 1

Reader 1	SR-PR	SL-PL	SR-PL	SL-PR	SR-SL	PR-PL
Statistic	7.1	8.8	5.0	9.2	−2.3	−1.9
*df*	54	52	52	54	54	52
*p*	<0.001	<0.001	< 0.001	<0.001	0.022	0.058
Mean difference	6.8	7.1	5.0	8.9	−2.1	−1.7
SE difference	0.9	0.8	1.0	0.9	0.9	0.9
95% CI	…	…	…	…	…	…
Lower	4.9	5.5	3.0	6.9	−3.9	−3.6
Upper	8.7	8.7	7.0	10.8	−0.3	0.05
Effect size	0.9	1.2	0.6	1.2	−0.3	−0.2
Lower	0.6	0.8	0.3	0.8	−0.5	−0.5
Upper	1.2	1.5	0.9	1.5	−0.04	0.008

**Table 3:** Comparisons of means of DBRN angle in different sides and positions of the forearm for Reader 2

Reader 2	SR-PR	SL-PL	SR-PL	SL-PR	SR-SL	PR-PL
Statistic	6.4	9.6	6.1	8.0	−2.3	−0.7
*df*	54	52	52	54	54	52
*p*	< 0.001	< 0.001	< 0.001	< 0.001	0.020	0.431
Mean difference	6.4	7.8	5.6	8.6	−2.2	−0.8
SE difference	0.9	0.8	0.9	1.0	0.9	1.0
95% CI	…	…	…	…	…	…
Lower	4.4	6.1	3.7	6.5	−4.1	−2.8
Upper	8.4	9.4	7.4	10.7	−0.3	1.2
Effect size	0.8	1.3	0.8	1.0	−0.3	−0.1
Lower	0.554	0.9	0.5	0.7	−0.5	−0.3
Upper	1.1	1.6	1.1	1.4	−0.05	0.1

There were more convergent nerve deflections in maximal pronation (Reader 1: 91/108 [84.2%] and Reader 2: 82/108 [75.9%]). More divergent deflections were reported in maximal supination (Reader 1: 72/110 [65.4%], Reader 2: 74/110 [67.2%]). The neutral deflection was observed in maximal pronation on the left side in 1 case for Reader 1 and 4 for Reader 2.

In pairwise comparison, 93 out of 108 angles (86.2%) had a more convergent deflection in maximal pronation than in maximal supination for both readers (mean in pronation, Reader 1: 174.4°, Reader 2: 175.0°, and mean in supination, Reader 1: 181.4°, Reader 2: 182.1°). Only 15 out of 108 angles (13.8%) had a more convergent angle deflection in maximal supination.

On the right side (dominant side) (*N* = 12 in consensus), the inverted pattern of more convergent angle deflection in maximal supination was mainly found in those engaged in upper limb workouts (*N* = 7; 87.5%, in consensus), doing exercises with ≥ 5 kg of weight. On the left side (4 cases in consensus), cases with the inverted pattern were less frequent than on the right. There was an association with limb workout in one participant (25%), and coincidently, this person was left-handed and had the same inverted pattern on the right side.

### Interobserver agreement

ICC for the interobserver agreement was very good in phase 1 (SR: *r* = 0.87, *p* < 0.001, PR: *r* = 0.95, *p* < 0.001, SL: *r* = 0.88, *p* < 0.001, PL: *r* = 0.93, *p* < 0.001) and in phase 2 (SR: *r* = 0.90, *p* < 0.001, PR: *r* = 0.92, *p* < 0.001, SL: *r* = 0.92, *p* < 0.001, PL: *r* = 0.93, *p* < 0.001).

### Intraobserver agreement

ICC for the intraobserver agreement was very good for Reader1 (SR: *r* = 0.96, *p* < 0.001, PR: *r* = 0.93, *p* < 0.001, SL: *r* = 0.92, *p* < 0.001, PL: *r* = 0.96, *p* < 0.001) and Reader 2 (SR: *r* = 0.94, *p* < 0.001, PR: *r* = 0.95, *p* < 0.001, SL: *r* = 0.94, *p* < 0.001, PL: *r* = 0.93, *p* < 0.001).

### DBRN angles and correlations

Seven cases were excluded from this part of the analysis: five due to disagreements greater than the mean angle and two due to missing data in the pronation on the left side. There was no statistically significant correlation between the DBRN angles and the differences between the angles in supination and pronation (net ROM) and age, sex, weight, height, BMI, % muscle mass, and % body fat. In addition, there was no statistically significant correlation between those angles and the net ROM and sports activities with the upper limb, hours of computer work, and rotational work with the forearm. One exception was SR for Reader 1 (*p* = 0.046).

## Discussion

This study evaluated the effect of maximal pronation and maximal supination of the forearm on the longitudinal alignment of the DBRN at the most frequent site of nerve impingement. We identified patterns of the DBRN deflection at the level of the SASM and explored our initial hypothesis regarding nerve alignment in pronation. Our results demonstrated DBRN convergence towards the SASM in maximal pronation in more than 75% of the participants and divergence in maximal supination in more than 65%. In addition, we identified that the DBRN has, on average, a ROM of approximately 7° of deflection in the long axis.

Traditional US markers for peripheral nerve abnormalities include cross-sectional area, nerve swelling ratio, side-to-side cross-sectional area asymmetry, and changes in echogenicity [9, 13]. However, nerve neurodynamic is gaining recognition as an essential part of imaging evaluation, in addition to nerve morphology and structural changes [9, 14, 15]. Despite that, few reports evaluated the neurodynamic of the radial nerve and its branches with HRUS [16–18]. Martinoli et al. commented that the PIN (described as DBRN in our study) appeared to follow an angulated course at the arcade of Frohse, which should not be mistaken for a pathologic finding on ultrasound [16]. We agree with this statement, and in our study, we went further, quantifying the nerve’s angulation and demonstrating the existence of different patterns of nerve deflection with forearm rotation. Chen and Wang demonstrated the clinical application of forearm pronation and supination with HRUS in a case report of supinator syndrome [17].

Therefore, to the best of our knowledge, we were the first prospective observational study to investigate by imaging and in an in vivo human cohort the long-axis angulation of the DBRN in the SASM. It is tempting to hypothesize that a higher deflection angle would have implications on DBRN entrapment. However, considering several confounders, our study design does not support this assumption. For future studies, interesting insights can be provided by comparing the results of our normal population with a group of normal athletes and patients with DBRN entrapment syndrome.

Despite that, another hypothesis can be made based on our data, which may shed light on the pathophysiology of the DBRN entrapment syndromes. We consider the concept proposed by Shacklock that joint angulation increases the length of the nerve bed on the side of the axis of rotation that opens [19] and the engineering theory that when an isotropic rod bends, the outside material is tensioned and the inside is compressed [20]. We hypothesize that the DBRN lengthens in most cases on the deep side (furthest from the SASM) in pronation and on the superficial side (closer to the SASM) in supination. Therefore, both sides of the nerve will be under some degree of tension with full forearm ROM, particularly under the extremes of sports activities, such as baseball pitching, racket sports, and overhead throwing in football. Knowledge of the neurodynamics may have implications in sports practice and training programs. For instance, our data support injury prevention by reducing the number of repetitions of forearm pronation and supination, which reduces friction and tension on the deep and superficial surfaces of the DBRN. Considering our study and the bulk of information in the literature [2, 5], special attention should be given to the reduction of overload in pronation during athletic training regimens.

Factors traditionally associated with entrapment of the DBRN are the arcade of Frohse, *i.e.,* a SASM with tendinous consistency as opposed to muscular or membranous, and the increased pressure under the SASM in pronation [1–5]. However, more than this is needed to fully explain the dynamic nature of the entrapment of the DBRN at the level of the SASM. For instance, according to a recent meta-analysis, the arcade of Frohse is commonly found in adults, with a pooled prevalence of approximately 66% in this population [1]. On the other hand, increased pressure under the SASM seems to be a physiological finding, like the increased intrathoracic pressure observed with the Valsalva maneuver [21]. We speculate that there is a multifactorial nature of the dynamic entrapment of the DBRN, with our findings representing the missing piece of the puzzle. In summary, we believe that to trigger clinical symptoms of entrapment, in addition to anatomical (arcade of Frohse) and physiological factors (increased pressure in pronation at the SASM level), supraphysiological biomechanical stress on the nerve (repetitive flexion and friction) must be added, increasing the local level of neuroinflammation.

In our normal population, systemic biometric features, sports activities with the upper limb, hours of computer work, and forearm rotation during work showed no correlation with the DBRN angles and the net ROM. However, some characteristics of our population should be considered for comparison with future studies. Although most of our participants (61.8%) were engaged in sports activities with the upper limb, none were at the professional level or in preparation for a competition. In addition, rotational movements at work were reported by a minority of participants (25.4%), and in no case was there any association with continuous and repetitive heavy activities or the use of machinery. The majority of participants, i.e., 92.7%, were right-handed, a proportion very close to that estimated for the general population (81.9 to 90.7%) [22]. Hand dominance and muscle tension may have accounted for more positive deflections in the dominant right side in supination compared to the left side.

Our study had a very good inter and intraobserver agreement in assessing the DBRN angle, which we attributed to several factors, such as readers’ experience and ROI selection. Despite that, we observed a few discrepancies in the angle measurements, illustrating potential pitfalls with this technique. These challenges were related to a low amount of echogenic perineural fat associated with muscle hypertrophy (*N* = 3), adjacent vessel simulating the nerve path (*N* = 1), and nerve branching (*N* = 1). The analysis can be facilitated in the real world with the use of a cine clip to review the images, by correlation with the short axis of the nerve, and with the aid of Doppler. In addition, knowledge of positional changes in the DBRN alignment is important for the radiologist to avoid pitfalls during imaging interpretation and interventional procedures.

The study has several limitations. First, we did not consider the longitudinal excursion of the nerve with movements of other parts of the body [19, 23]. However, to mitigate the longitudinal nerve excursion, we partially immobilized the participants’ hands and wrists in the neutral position and oriented them to keep their heads straight, avoiding side rotation. It is worth mentioning that DBRN excursions can also occur in the transverse plane, and other nerve deflections may happen with movement. However, their dedicated analysis is out of the scope of our study. These other components of DBRN neurodynamics were at least partially incorporated into our angle analysis, as the transducer was positioned in a way to visualize the longest longitudinal segment of the nerve, regardless of forearm position and positional nerve changes. Second, our population comprised South Americans, primarily Brazilians, and, therefore, results may vary in different populations, with different biometrical features, social behaviors, and distinct physical, work, and sports activities. Third, the nerve was only assessed at the SASM, and the neurodynamics of the nerve at other areas have also been implicated in entrapment syndromes. Furthermore, the ROI used in the study focused on one specific nerve area, but other regions of the nerve and adjacent structures may have an additional role in the angulation and entrapment of the DBRN [24].

In conclusion, our study provides additional information on the neurodynamics of the DBRN at the level of the SASM. A dominant pattern of angulation of the DBRN with a more convergent deflection in maximal pronation and divergent deflection in maximal supination was observed. Knowledge of the patterns of positional changes of the DBRN is important for the radiologist during imaging interpretation and interventional procedures. It may also have implications in sports practice and training programs.
